# Trends and Effectiveness of ICT Interventions for the Elderly to Reduce Loneliness: A Systematic Review

**DOI:** 10.3390/healthcare9030293

**Published:** 2021-03-07

**Authors:** Hee Kyung Choi, Seon Heui Lee

**Affiliations:** Department of Nursing Science, College of Nursing, Gachon University, Incheon 21936, Korea; hcgsf0910@naver.com

**Keywords:** aging, ICT-based care service, social isolation, loneliness, systematic review

## Abstract

Elderly people are sensitive to loneliness, which may contribute to mental and physical health, serious illness, and increased mortality. This study investigates the development trend of information communication technology (ICT) interventions designed for the elderly to reduce loneliness and synthesize its effect. We searched relevant articles on 23 May 2020 using three databases: Ovid-Medline, Ovid-EMBASE, and the Cochrane library. Data extraction and quality assessment were independently performed by two authors. The development is changing from animal robots to online social platforms and from simple emotional support to a multifaceted system that promotes social participation, cognition, physical activity, and nutrition. Our systematic review reported that ICT interventions are being developed to alleviate loneliness and increase social participation. Our study revealed an increase in the use of ICT interventions among the elderly and a positive change in their attitude toward ICT interventions. ICT interventions in the field of nursing should continue to be developed in the future to meet social, health, and safety needs. In the context of coronavirus disease 2019 (COVID-19), ICT interventions are needed to respond effectively to the needs of the elderly. This study is expected to provide basic knowledge for the development of ICT interventions for the elderly.

## 1. Introduction

According to the current population trend, the proportion of the elderly is expected to rise significantly [1]. Elderly people are sensitive to social isolation and loneliness, which may contribute to mental and physical health, serious illness, and increased mortality [2,3,4,5]. Consequently, reducing social isolation and loneliness in the elderly is an important topic in many countries [6,7]. To prevent the potential risks of social isolation and loneliness, it is crucial to develop social interventions for the age group [8].

The belief that face-to-face interaction is the standard of social participation has been challenged [9], and there is evidence that communication technology can establish feelings of connectivity [10]. Information communication technology (ICT) has emerged to improve the social participation and health conditions of the elderly by reducing social isolation and loneliness [11]. Specifically, there are general ICT interventions, such as the use of the Internet and computer training, and ICT interventions designed for the elderly. ICT interventions designed for the elderly have been developed to help them overcome technical barriers [12], while taking into consideration the interests and preferences of the elderly [12,13,14]. Many recent studies have also been developing ICT interventions, particularly those that follow a senior-centered design [15,16,17].

One study reported that the enjoyment of an ICT intervention designed for the elderly is higher than that of existing ICT interventions for everyone in the market, including traditional groups [12]. In previous studies, ICT interventions designed for the elderly have been reported to be effective in reducing social isolation and loneliness [18,19,20,21,22] by promoting social communication [23,24,25,26,27] and improving participation in physical activities [23,26] among the elderly. ICT interventions have also been accepted by the elderly [22,26] who have a positive attitude toward them, and are intended for daily use [25,28].

Several systematic reviews have examined the effects of ICT interventions on social isolation and loneliness in the elderly [11,29,30,31]. However, these studies reported general ICT interventions, such as the use of the Internet and computer training, not ICT interventions designed specifically for the elderly. Although several studies partially included ICT interventions designed for the elderly, they did not systematically examine the development trends. Moreover, these studies have not comprehensively summarized either the effect of reducing social isolation and loneliness or the satisfaction of using ICT interventions.

Therefore, a comprehensive systematic review including recently published literature needs to be performed to investigate the development trends of and summarize the effects of ICT interventions designed for the elderly to reduce social isolation and loneliness.

## 2. Materials and Methods

This systematic review was conducted to confirm the development trends and effectiveness of ICT interventions designed for the elderly according to the Preferred Reporting Items for Systematic Reviews and Meta-Analyses (PRISMA) guidelines.

### 2.1. Search Strategy

We searched relevant articles on 23 May 2020 using three databases: Ovid-Medline, Ovid-EMBASE, and the Cochrane library. To retrieve eligible articles, the following keywords were used: ((elderly OR older OR seniors OR aging), (information communication technology OR ICT OR social media OR robot OR virtual reality OR application OR online platform OR game OR computer system), and (social isolation OR loneliness OR social support OR social connect OR social interaction OR well-being OR social ties OR the quality of elderly life OR physical and cognitive training OR health care)). All of the search terms and Medical Subject Headings (MeSH) are presented in Appendix A.

### 2.2. Eligibility Criteria and Study Selection

Two authors of the current study (LSH and CHK) independently selected relevant studies based on predefined inclusion criteria: (a) Studies involving elderly people aged 60 or older; (b) Studies conducting ICT interventions designed for the elderly to reduce loneliness and social isolation; and (c) Studies including protocols and reviews to consider the latest ICT interventions. We excluded studies if they met the following criteria: (a) Posters, abstracts, animal studies, articles not published in English, and duplicated studies; and (b) Studies performing general ICT interventions such as general Internet use, SNS, video games, and others not designed for the elderly. Two reviewers independently screened the titles and abstracts based on the inclusion and exclusion criteria. Full texts of affiliated studies were examined by the same reviewers. The reliability was checked by two authors using Cohen’s kappa coefficient.

### 2.3. Data Items and Data Collection Process

Data extraction was performed independently and checked by two reviewers using a data collection form predefined by mutual agreement. We extracted some components of the population for ICT interventions, such as sample size, age, sex, subject criteria, and others. We also investigated the development trends and outcome measurements.

We extracted outcomes such as loneliness, social isolation, life satisfaction, social support, quality of life, other emotional responses, usage and usability, and attitudes toward ICT interventions. In case of loneliness indicators (University of California-Los Angeles Loneliness Scale, 20 to 80; Ando, Osada, and Kodama Loneliness Scale, 0 to 10; Short Form of University of California-Los Angeles Loneliness Scale; De Jong Gierveld Loneliness Scale, 0 to 6), lower scores of the social isolation tools (Friendship Scale, 0 to 24) represent a decreasing level of loneliness, while higher scores indicate alleviated levels of social isolation.

Regarding indicators such as life satisfaction (Satisfaction with Life Scale), social support (Duke Social Support Scale; Interpersonal Support Evaluation List, 6 to 36), and health-related quality of life (MOS 36-Item Short-form Health Survey, 0 to 100), higher scores represent a greater level of life satisfaction, social support, and health-related quality of life.

Concerning other affective responses, which are facial expressions (face scale, 1 to 20), moods (The Profile of Mood States, 0 to 4), well-being (Satisfaction with Life Scale; World Health Organization-Five Well-Being Index), comport, satisfaction, and happiness (1 to 5), exercise enjoyment (Physical Activity Enjoyment Scale), and self-efficacy (General Self-Efficacy Scale), higher scores indicated a more positive emotional status of the elderly. Additionally, indicators of salivary chromogranin A, 17-ketosteroid sulfate (17-KS-S) values, and the ratio of 17-ketosteroid sulfate/17-hydroxycorticosteroids (17-KS-S/17-OHCS) were examined, and lower scores indicated a lower level of stress.

As for usability indicators such as usefulness and usability (Technology Acceptance Questionnaire), higher scores and values meant that the ICT interventions were easy to use. For perceptions of ICT interventions and attitudes toward technology (5 to 25), higher scores meant more positive perceptions. Additionally, social cognition (Young Schema Questionnaire) and perceived vulnerability (Perceived Vulnerability Scale, 1 to 6) were examined, and lower scores of the indicators indicated more positive attitude toward ICT interventions.

### 2.4. Risk of Bias in Individual Studies

Quality assessment of the studies was performed by two authors using the Cochrane Risk of Bias tool (RoB) for randomized controlled trials (RCTs). The assessment consists of seven domains: random sequence generation (selection bias), allocation concealment (selection bias), blinding of participants and personnel (performance bias), blinding of outcome assessment (detection bias), incomplete outcome data (attrition bias), selective reporting (reporting bias), and others (other bias). Each domain of the RoB was evaluated with “low,” “high,” or “unclear” risk of bias. In case of disagreement, the two reviewers discussed and agreed on the outcome.

The quality of non-randomized studies was evaluated using the RoB for Nonrandomized Studies (ROBANS). The assessment tool involves six domains: the selection of participants, confounding variables, measurement of intervention (exposure), blinding for outcome assessment, incomplete outcome data, and selective outcome reporting. Further, each domain was scored as 2 (reported and adequate), 1 (reported but inadequate), or 0 (not reported).

## 3. Results

### 3.1. Study Selection

**Figure 1:**
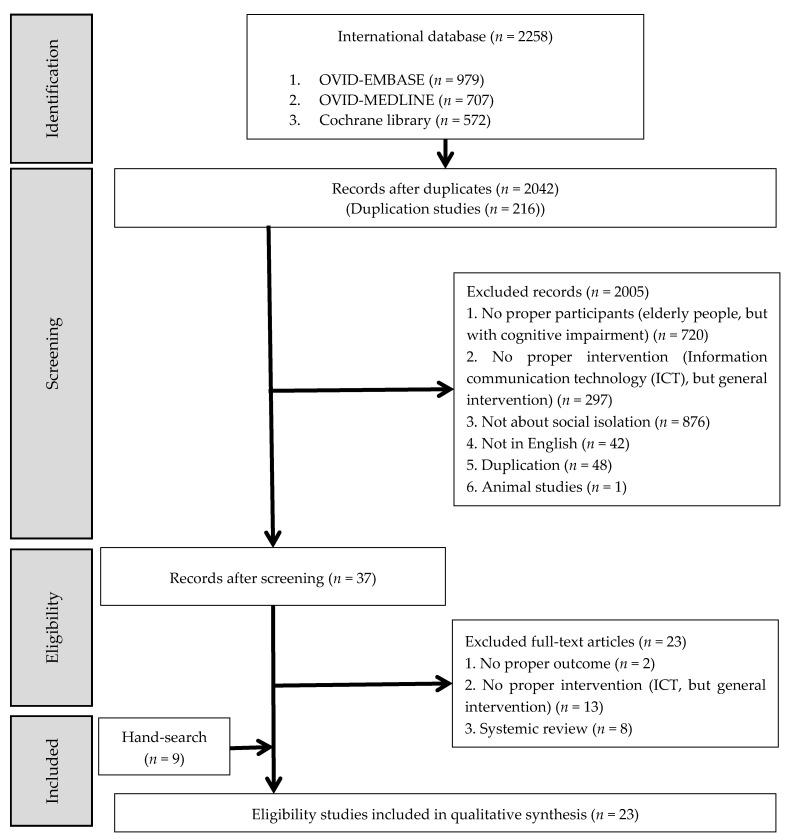
Flow chart of the study selection.

After full-text reviews, 14 articles were identified as appropriate for this study. Nine additional articles were found by hand search, and 23 studies were included in the final review process through discussion. Figure 1 illustrates the flowchart of the selection procedure. We checked the reliability using Cohen’s kappa coefficient (*k* = 0.98).

### 3.2. Characteristics of Included Studies

Table 1 summarizes the characteristics of the 23 studies, which included four randomized controlled trials, two non-randomized controlled trials, five before studies, three mixed methods, four in-depth interviews, one observational and qualitative study, two reviews, and two randomized controlled trial protocols. These studies searched on 23 May 2020 had been published between 2003 and 2019. The ICT interventions in the included studies were developed via seven studies from Europe, one from North America, five from South America, five from Asia, one from Africa, and two from multiple continents.

### 3.3. Quality Assessment

**Figure 2:**
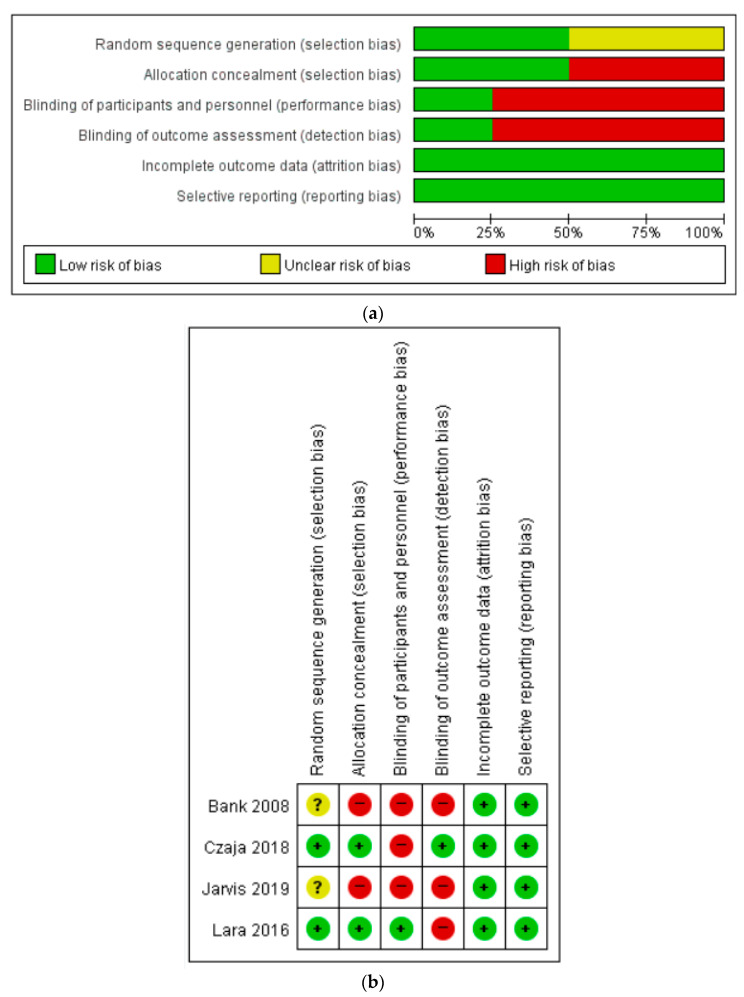
Quality assessment (**a**) Risk of bias graph, (**b**) Risk of bias summary.

Of the 23 selected studies, four were RCTs, and two were non-RCTs. For the four RCTs assessed using the RoB tool, the risk of attrition bias and reporting bias was low. Three of the four studies had a high risk of performance and detection biases. In selection bias, random sequence generation was high, and the allocation concealment was unclear in two of the four studies. The results of the quality assessment using the RoB tool are shown in Figure 2. For the two non-RCTs assessed using the ROBANS tool, the risk of selection of participants, confounding variables, measurement of intervention (exposure), incomplete outcome data, and selective outcome reporting was low. However, the risk of blinding for outcome assessment was high.

### 3.4. Development Trend

Table 2 provides a summary of the development trends of the included papers in the review. The types of ICT interventions designed for the elderly varied, so the trends in the papers were identified by categorizing them in order of time.

#### 3.4.1. Animal Robot

Five studies targeted animal robots that look like dogs or seals. The robot has a variety of sensors that can react to stimuli. Additionally, the robot has several needs, based on a diurnal rhythm [18,21]. Another robot expresses emotions and recognizes language through communication. Consequently, interaction with the robot has psychosocial effects on the elderly [27,32,33].

#### 3.4.2. Humanoid Agent

Two studies reported software humanoid animated agents that assess the elderly’s affective state and the number of steps per day. Daily conversations with them ease the sense of social isolation, and appropriate healthy feedback motivates the elderly to engage in physical activities. The agents present a graph of their progress, discuss obstacles, and negotiate a goal for the following day [22,23].

#### 3.4.3. Mobile Robot

Four studies presented assistive mobile robots that allows users to control navigation and facilitate social interaction by providing video communication with other people [34]. Therefore, the elderly can reduce loneliness, and caregivers can remotely control the robot and observe the daily life of the elderly. Additionally, some robots carry small objects, measure vital parameters [25,28], detect danger, and help carry out daily activities at home [13].

#### 3.4.4. Exercise Game

Three studies presented an exercise game that is a combination of videogames and exercise. The games allow communication with others, which helps to reduce loneliness during exercise [12,15,35]. Considering the interests and physical conditions of the elderly, an exercise game was designed to achieve motivation and enjoyment.

#### 3.4.5. Interpersonal Communication

Two studies targeted social applications [24,36]. One app allows interaction through photos, audio, videos, and messages using iPads. The interface offers large touch icons for the elderly [36]. These apps enable the elderly to have contact with each other in their homes and reduce loneliness.

#### 3.4.6. Online Social Platform

Seven studies targeted online social platforms that provide easy access to information sources and opportunities to communicate. These platforms promote a lifestyle for the elderly, including physical activities, cognitive function, a safe environment, and emotion. These platforms also include various functions such as self-monitoring, calendars, photos, games, and online help [14,19,20,26,37]. Reflecting the opinions of the elderly, two of seven studies are currently underway [16,17].

### 3.5. Effectiveness of ICT Interventions Designed for the Elderly

Of 23 selected articles, 14 measured the effectiveness of the ICT interventions designed for the elderly. The results are presented in Table 3. Eight reported the effectiveness of loneliness and social isolation. One examined life satisfaction, two measured social support, one reported quality of life, and eight examined other affective responses. Six reported use and usability, three showed attitudes toward ICT interventions, and two reported others including nutrition and physical activity.

#### 3.5.1. Loneliness and Social Isolation

Eight studies included loneliness and social isolation. There was a decrease in loneliness in the intervention group (3.33 ± 2.16 to 1.00 ± 1.26; *p* = 0.07) [21] as compared to the passive group (3.57 ± 6.10 vs.−0.8 ± 2.77; *p* = 0.13) [22]. Additionally, there was a significant decrease in loneliness among those in the intervention group (3.53 ± 1.3 to 1.38 ± 1.33; *p* < 0.001) as compared to those in the usual care group (3.59 ± 1.23 to 4.00 ± 1.32; *p* = 0.064) [20], and there was a significant decrease in loneliness and social isolation (*p* < 0.01) [19]. There were no statistically significant differences in loneliness among those in the intervention group (*p* > 0.05), but the outcomes were similar to those of the usual care group [12,18,23]. However, there were no significant changes in loneliness before and after the study (*p* > 0.05) [36].

#### 3.5.2. Life Satisfaction

One study included life satisfaction. It significantly differed between the three groups (*p* = 0.234) because of the small sample size, family conflicts, and activities provided to control groups [12].

#### 3.5.3. Social Support

Two studies included social support. It was improved and maintained after intervention (*p* < 0.01) [19]. However, there were no significant changes (*p* > 0.05), but the elderly answered in an interview that the intervention increased positive mood and self-efficacy [36].

#### 3.5.4. Quality of Life

One study included quality of life. The intervention served to maintain and improve the quality of life after the intervention (38.63 ± 38.86 to 75.00 ± 41.83; *p* = 0.03). In the activity evaluation, the scores of the last activity significantly increased compared to that of the initial activity [21].

#### 3.5.5. Other Affective Responses

Eight studies included other affective responses. There were positive emotional responses. Scores on the face scale and moods increased after the intervention (*p* < 0.05) [33]. Comfort (4.59 ± 0.80 vs. 4.33 ± 0.85), satisfaction (3.95 ± 1.08 vs. 3.14 ± 1.26), and happiness (3.89 ± 0.90 vs. 3.26 ± 1.26) improved compared to the passive group (*p* < 0.05) [22]. Scores of exercise enjoyment were higher than those in other groups (24.4 ± 0.65 vs. 22.0 ± 0.65; *p* > 0.05) [12]. Emotional words (1.40 ± 0.55 to 2.40 ± 0.55; *p* = 0.03), amount of speech (1.20 ± 0.45 to 2.50 ± 0.55; *p* = 0.04), and satisfaction (1.60 ± 0.55 to 3.00 ± 0.00; *p* = 0.04) increased after the intervention. Moreover, the reaction to stress eased after the activity. Salivary chromogranin A (CgA) decreased after intervention (1.14 ± 0.63 to 0.94 ± 0.74; *p* < 0.01) [21]. The hormone values of 17-KS-S (1.00 ± 0.51 to 1.41 ± 1.09) and the 17-KS-S/17-OHCS ratio (0.18 ± 0.08 to 0.26 ± 0.09) improved after the activity (*p* < 0.05) [32,33]. However, self-efficacy was lower than other groups (*p* > 0.05) [12], and well-being was not significantly different between the intervention and usual care groups (*p* > 0.05) [20,23]. In contrast, well-being increased significantly (*p* < 0.05) [19]. Since the elderly performed the intervention alone, limited social interaction occurred [12,23].

#### 3.5.6. Usage and Usability

Six studies included usage and usability. The use of ICT interventions has increased [20,23]. Attachment to intervention increased [18], and the density of objective social networks and average time spent increased [27]. Moreover, the interviewed elderly responded that ICT interventions were easy to use and helpful [19,23,28].

#### 3.5.7. Attitude toward ICT Interventions

Three studies included attitudes toward ICT interventions. Perceptions of ICT interventions were mostly positive [23]. Social cognition about abandonment, defectiveness, emotional deprivation, mistrust, and social isolation of the intervention group (83.53 ± 19.3 to 52.62 ± 15.99; *p* = 0.008) significantly decreased compared to the usual care group (73.82 ± 29.05 to 78.00 ± 14.77; *p* = 0.275) [20]. Attitudes toward technology changed positively (*p* < 0.01) and perceived vulnerability decreased in the intervention group (*p* < 0.001) [19].

#### 3.5.8. Others: Diet Management, Weight Control, and Physical Activity

Two studies included other interventions such as diet management, weight control, and physical activity. The Mediterranean diet score (4.7 to 4.6) was relatively higher than that of the usual care group (3.8 to 3.8). The body weight (−0.6 vs. −0.3 kg) and waist circumference (−0.9 vs. −0.4 cm) had a smaller reduction than the usual care group. Regarding physical activity, all physical outcomes were similar to those of the usual care group [26]. The increase per week in mean steps walked (411.1 vs. 83.9) was more than that in the standard care group (*p* = 0.004) [23].

## 4. Discussion

In our systematic review, we examined 23 studies related to ICT interventions designed for the elderly to reduce loneliness and social isolation. To the best of our knowledge, our study is the most updated and comprehensive systematic review conducted so far and the only one that analyzes the development trends and effects of ICT interventions designed for the elderly. The principal finding of this systematic review is that the trend of development is changing from animal robots to online social platforms and from simple emotional support to a multifaceted system that promotes social participation, cognition, physical activity, nutrition, and sleep. Secondly, our results showed that ICT interventions for the elderly are being developed to alleviate loneliness among the elderly and increase social participation. Thirdly, our systematic review revealed that the elderly increased their use of ICT interventions, and have a positive attitude towards it.

In our study, we examined the development trends of ICT interventions, which is shifting from animal robots to online social platforms especially developed for the elderly. Initially, animal robots were designed for entertainment, communication, and mental therapy [27]. However, the form was evolving from an animal robot that soothes loneliness to online social platforms with more comprehensive functions. Another type of humanoid agent, which appeared as a person on the screen, provided an effective interface modality for the elderly and became a conversational partner and an exercise advisor [23]. Mobile robots assist the elderly in activities of daily living and social interaction, and offer multiple functionalities, such as navigation; fetching, and carrying small objects; measuring vital parameters, providing reminders, a calendar, and interpersonal communication [25]. Others are ICT interventions in exercise games and interpersonal communication technology. The exercise game form allows the elderly to overcome barriers to low exercise adherence by increasing the players’ enjoyment [12] and reducing the risk of social isolation [15]. Interpersonal communication technology can create social connections, helping alleviate social isolation and loneliness [36]. The next step was to create online social platforms. Recently developed online social platforms promoted healthy eating, physical activity, and meaningful social roles [14,17,19]. These services can also support more comprehensive support than previous types of ICT interventions. They can improve the quality of life and help maintain their independence, including improving loneliness and isolation, preventing falling, and managing medications, driving, and transportation [14]. Additionally, they can facilitate active and healthy aging, and prevent cognitive impairment, frailty, depression, anxiety, social isolation, poor sleep quality, and falls [17].

Looking at the research results included in this review, the needs of the elderly have been identified, and efforts have been made to satisfy them. The fundamental needs of the elderly were health needs such as cognitive function or chronic disease management, safety needs such as fall prevention, and social needs such as social participation to reduce loneliness. Therefore, future developments should be proposed not only in emotional care, including loneliness and social isolation but also in a multifaceted way. The results of other systematic reviews [8,11,29,30,31,38,39,40] do not systematically organize the development trend as in our study. However, they suggest that the ICT interventions should be developed to promote affection, health, and safety of the elderly, as shown in our results. The roles of technology should meet the various needs of the elderly. In conclusion, ICT interventions designed for the elderly in the field of nursing should continue to be developed to meet social, health, and safety needs.

Secondly, considering the effects of ICT interventions designed for the elderly, most studies have examined the general effectiveness in improving loneliness, life satisfaction, social support, quality of life, health outcome, and other affective responses compared to real animals or usual care. Some systematic reviews have also identified the effectiveness of reducing social isolation and loneliness and increasing social networks as in our study [8,11,30,40]. Other systematic reviews that reported the social support result also showed a good effect, consistent with our results [38,39]. Another systematic review reported health outcomes that were consistent with our study [31].

However, several studies reported no statistically significant difference between the intervention and control groups regarding loneliness. In those studies, the elderly performed the intervention alone with no supervision, so limited social interaction occurred [12,23]. It determined that to increase the effectiveness of the intervention, compliance should be increased through continuous monitoring. In other cases, although there was no statistical difference in social support, the elderly answered in an interview that the intervention increased positive mood and self-efficacy [36]. The reason previous studies were not statistically significant is because of the small sample size, other life events such as family conflicts, and activities provided to the control group [12]. In terms of the effectiveness of ICT interventions on affect, most studies reported positive emotional responses, such as mood, facial expression, emotional words, amount of speech, comfort, happiness, satisfaction, exercise enjoyment, well-being, and stress reduction [12,19,21,22,27,32,33]. However, some studies using small samples reported that well-being did not significantly change after intervention [20,23]. Although we comprehensively analyzed papers worldwide, because studies had a short intervention interval and a small number of participants who lived alone, further studies designed with a large sample size are required.

The elderly increased their use of ICT interventions, and there was a general increase in positive attitudes toward computers. Most studies reported that the elderly would like to continue using the system, and a significant increase in the frequency of usage demonstrated that elderly use the system comfortably and with minimal training. The majority of users indicated that the intervention was easy to use. To satisfy the needs of the elderly for healthy social participation, administrative care, and safety using ICT, the following conditions were met: affordability, usability, and accessibility. According to other previous study results, it is reported that affordability, usability, and accessibility are the main elements of non-face-to-face services for the elderly [41,42,43]. In our systematic review, there was substantial evidence that the usability of non-face-to-face interventions for the elderly, despite many concerns, has improved. In the future, additional studies that can deal with not only the usability of online interventions but also their affordability and accessibility should be conducted.

In the context of coronavirus disease 2019 (COVID-19), which has become another barrier for face-to-face interaction, it is increasingly difficult to implement offline social services for the elderly through the community care system. With the lack of offline services, the physical condition of the elderly is in greater danger of worsening, which can cause high levels of stress, anxiety, depression, loneliness, and social isolation. Thus, to respond effectively to the needs of the elderly during the COVID-19 crisis, innovative online solutions based on ICTs are needed for personalized interventions [44]. In the future, as the importance of non-face-to-face service increases, this study is expected to provide basic knowledge for the development of such interventions for the elderly.

We identified the development trend and effectiveness of ICT interventions designed for the elderly. However, this study has several limitations. First, most studies in this systematic review varied widely in the methodology regarding the ICT interventions conducted. However, we included reviews or protocols to consider the latest ICT interventions. Second, heterogeneous interventions make comparisons difficult, and it is challenging to draw conclusions. Therefore, the results should be interpreted with caution. Third, more studies are needed to examine the effectiveness of ICT interventions. Therefore, evaluating the effectiveness of ICT interventions requires more randomized clinical trials with large-scale samples and comparative studies. Finally, the outcome measurements were often reported in different ways, which made meta-analysis impossible for selected studies.

## 5. Conclusions

The development trend of ICT interventions for the elderly is gradually changing from animal robots that provide emotional support to the online social platforms that can promote social participation, cognition, physical activity, nutrition, and sleep. Our research showed that ICT interventions designed for the elderly, compared to real animals or usual care, have proved to be effective in improving loneliness, life satisfaction, social support, quality of life, and other affective responses. Based on this study, ICT interventions designed for the elderly should continue to be developed in the nursing field to meet social, health, and safety needs.

Previous studies have reported that the key elements of ICT interventions for the elderly are affordability, usability, and accessibility [41,42,43]. Our study revealed the improved usability of ICT interventions for the elderly. In the future, studies should be conducted to address the usability of ICT interventions as well as their affordability and accessibility. With the increasing importance of non-face-to-face services in the context of COVID-19, we expect our systematic review to be fundamental knowledge for the development of ICT interventions for the elderly.

## Figures and Tables

**Table 1:** General characteristics of selected articles.

Type	Information Communication Technology (ICT)	Reference	Study Design	Development Country	Effectiveness Analysis
Animal robot	AIBO	[18]	RCT	USA	√
		[21]	Before after study	Japan	√
	Paro	[32]	Before after study	Japan	√
		[27]	Before after study	Japan	√
		[33]	Before after study	Japan	√
Humanoid agent	Relational agent	[23]	Non RCT	USA	√
	Conversational agent	[22]	Mixed methods	USA	√
Mobile robot	Mobile remote presence systems	[34]	Semi-structured interview	India, USA	
	Assistive telepresence robot	[25]	Mixed methods	Macedonia	√
		[28]	Mixed methods	Macedonia	√
	Astro robot, Buddy robot, Socialization application	[13]	In-depth interview	Italy	
Exercise game	Age invaders	[35]	Observational and qualitative study	Japan	
	Wii exergame	[12]	Non RCT	Finland, Singapore, and Japan	√
	Social Bike	[15]	Review	Italy	
Interpersonal communication	Real video communication	[24]	Interview	Finland	
	iPad-based communication app	[36]	Before after study	Canada	√
Online social platform	Elder Tree	[14]	RCT protocol	USA	
	PRISM	[19]	RCT	USA	√
		[37]	RCT protocol	USA	
	LEAP	[26]	RCT	England	√
	Palette V2 platform	[16]	Focus group interview	Netherlands, Romania, and Switzerland	
	LI-CBT	[20]	RCT	South Africa	√
	My-AHA	[17]	Review	European union	

AIBO = Artificial Intelligence Robot; RCT = Randomized controlled trial; Non RCT = Non-randomized controlled trial; USA = United States of America; PRISM = The Personal Reminder Information and Social Management; LEAP = Living, Eating, Activity, and Planning through retirement; LI-CBT = Low-intensity Cognitive Behavior Therapy; My-AHA = My Active and Healthy Ageing.

**Table 2:** Development trend.

Type	Information Communication Technology (ICT)	Country	Subject	Objective	Main Function and Method of Use	Expectation of Effectiveness
Animal robot	AIBO [18,21]	Japan	Elderly people in nursing homes	To maintain and improve the quality of life among elderly patients in nursing home	A dog-shaped robot has several functions including autonomy, emotional expression, learning its name, recognizing languages. The robot can learn and grow vicariously through communication.	It can be used as an alternative to animal therapy in the aseptic room, ICU, nursing homes for the elderly persons and children’s ward, and as psychosocial therapy.
	Paro [27,32,33]	Japan	Elderly people and nurses in a day service center	To provide psychological, physiological, and social effects in human beings through interaction	A seal-shaped robot has tactile, vision, auditory, and posture sensors. The robot reacts to sudden stimulation. It has a diurnal rhythm and several spontaneous needs, such as sleep, based on this rhythm.	Interaction with Paro has psychological, physiological, and social effects on elderly people.
Humanoid agent	Relational agent [23]	USA	Elderly people	To establish social-emotional relationships with users	Relational agents are software humanoid animated agents that appear as a person on the screen. The daily conversation involves the number of steps on the previous day, showing a graph of their progress, providing feedback, discussing obstacles, and negotiating a goal for the following day.	It provides an effective automated health educator with unbounded patience and empathy for these patients.
	Conversational agent [22]	USA	Elderly people	To provide social support and wellness counseling to isolated elderly people in their homes	The agent that appears as a person on the screen assesses the elderly person’s affective state and provides appropriate feedback designed to promote physical activity and help combat depression.	It assesses affect in users through dialogue and provides potential treatment for affect related disorders.
Mobile robot	Mobile remote presence systems [34]	India, USA	Community-dwelling elderly people	To foster social interaction between people	It allows the users to control navigation and webcam angles to enhance one’s sense of remote presence. It can be used in the workplace or home.	It assists the elderly in improving social connectivity with remote people.
	Assistive telepresence robot [25,28]	Macedonia	Elderly people and caregivers in nursing homes	To improve the well-being of the elderly by supporting daily activities independently	The robot permits various interactions in a remote environment, like navigation, fetch and carry small objects, measuring vital parameters of an elderly person, and providing reminders, a calendar, and interpersonal communication. The robot can be remotely controlled by a caregiver.	It assists the elderly in daily activities and provides video contact with other people.
	Astro Robot, Buddy Robot, Socialization application [13]	Italy	Elderly people	To promote well aging in daily life	It helps users to stand up and walk, contributing to improving walking capacity. It helps users to promote independence by detecting potential risks, in addition to inducing conversation rehabilitation.	It is more useful for independent living, considering demographic shift, economic constraint, and societal change.
Exercise game	Age invaders [35]	Japan	Grandparents and grandchildren	To emphasize inter-generational social interaction	Age invaders is a novel interactive inter-generation social-physical game. Two teams consist of one elderly and one young player. According to the players’ age, the game will be automatically balanced by the system. Parents can interact by using energy or barrier items for the player.	It is a good platform for grandparents to interact in real time with their grandchildren.
	Wii exergame developed for study [12]	Finland, Singapore, and Japan	Community-dwelling elderly people	To reduce loneliness, increase quality of life, and improve self-efficacy for the elderly	Exergame is a combination of videogames and exercise. Considering the interests and physical conditions of the elderly, five new exergames have been designed to enhance their well-being.	It promotes exercise adherence, reduces the barriers to digital technologies, and improves psychosocial well-being.
	Social Bike [15]	Italy	Elderly people at home	To reduce the risk of falling and promote social participation	All the users ride a virtual game-connected bike in their home and talk to others in their group. They wear sensors and maintain the cycling velocity. They push the button when the randomly assigned target appears. The cognitive task is represented by a score.	It encourages the elderly to converse with other elderly people and promotes their social participation.
Interpersonal communication	Real video communication [24]	Finland	Elderly people living at home	To have technological solutions that enhance health and quality of life	The system allows the elderly to contact each other in their homes. It enables users to hear about new things such as a range of gymnastics, memory training, and health education.	It enables the elderly to remain in contact with each other easily and to improve their quality of life while living at home.
	iPad-based communication app [36]	Canada	Elderly people in residential care	To support asynchronous communication among the elderly with family and friends	The app allows users to send and receive photos, audios, videos, and text messages. The interface offers large non-textual touch icons; not typing, only swiping or tapping.	It is an opportunity for social connectedness, helping alleviate social isolation and loneliness.
Online social platform	Elder Tree [14]	USA	Elderly people and their family caregivers	To support social connectedness, driving, caregiving, medication management, and fall prevention	Elder Tree is a multifaceted intervention with interacting services. Elder Tree comprises the following modules: “Learning,” “Communication,” and “Self-assessment Using tools.”	It reduces the physical, emotional, and financial burdens of the elderly and their families.
	PRISM [19,37]	USA	Elderly people who live independently in the community	To provide support and reduce isolation among the elderly	It is a robust support system with training and instructional support. PRISM comprises the following modules: “Internet access,” “Resource guide,” “Classroom,” “Calendar,” “Photo,” “E-mail,” “Game,” and “Online help.”	It reduces loneliness among the elderly. It increases technology self-efficacy.
	LEAP [26]	England	Elderly adults of retirement age	To promote healthy eating, physical activity, and meaningful social roles	LEAP comprises the following modules: “Time,” “Moving more,” “Being social,” “Eating well,” “Diary,” and “Dashboard.”	It helps the elderly to participate in the intervention to improve diet, physical activity, and social connections that are expected to enhance healthy aging.
	Palette V2 platform [16]	Netherlands, Romania, and Switzerland	Elderly people	To support seniors using an online support platform	The online platform matches elderly people that have similar interests and encourages participation in social activities. Design concepts comprise the following as: “Facilitate human contact rather than substituting it,” “Facilitate connection between users,” “Focus, without limiting, on a local operating scale,” “Create confidence,” “Respect the privacy of personal information,” and “Provide a content rating.”	It makes seniors attach importance to the trustworthiness and the need to maintain social ties.
	LI-CBT [20]	South Africa	Elderly people in residential care	To target maladaptive cognitions in elderly people experiencing loneliness	The intervention has distinct phases, requiring WhatsApp. Technology acceptance is developed through activities. Psycho-education is delivered on facts underlying loneliness. Individualized messages aimed at countering cognitions are sent. The weekly face-to-face Help Desk is continued.	It is effective in adjusting maladaptive social cognitions and reducing loneliness in elderly people.
	My-AHA [17]	European Union	Elderly people with pre-frailty symptoms	To improve physical activity, cognitive function, psychological state, nutrition, sleep, and well-being in the elderly	Physical, behavioral, psychological, cognitive, and nutritional data used by an ICT platform are retrieved from the user’s wearable sensors. Personal interventions generated by the system minimize health-related risks.	It monitors their current status and reduces the risk for frailty by following a tailored intervention.

ICT = Information communication technology; AIBO = Artificial Intelligence Robot; USA = United States of America; PRISM = The Personal Reminder Information and Social Management; LEAP = Living, Eating, Activity, and Planning through retirement; LI-CBT = Low-intensity Cognitive Behavior Therapy; My-AHA = My Active and Healthy Ageing.

**Table 3:** Effectiveness of information communication technology (ICT) intervention for the elderly.

Type	ICT	Study Design	Sample Size *N* (I/C)	Age (Mean ± SD)	Female *N* (%)	Subject Criteria	Outcome Measurements	Outcome
Animal robot	AIBO	RCT [18]	38(12/13/13) - AIBO 12 - Dog 13 - No animal-assisted therapy 13	N/A	N/A	Elderly patients living in long term care facilities MMSE ≥ 24 No allergies to dogs or cats UCLA loneliness scale ≥ 30 No history of psychiatric disease	Loneliness (UCLA loneliness scale)	There was no statistically significant difference between the AIBO and Dog groups.
							Attachment to pets (MLAPS)	The results revealed significant attachment in both the AIBO and the Dog groups. General attachment (I/C: 23.1 ± 1.6/25.5 ± 1.4) People substituting (I/C: 13.4 ± 1.9/16.5 ± 1.7) Animal rights (I/C: 10.3 ± 0.91/12.6 ± 0.71)
		Before after study [21]	5	68.2 ± 3.7	5(100)	Elderly people in a nursing home Having good cognitive function	Loneliness (AOK)	Scores obtained after the activity were lower than before the activity (3.33 ± 2.16 to 1.00 ± 1.26; *p* = 0.07).
							HR-QoL (SF-36)	Role-Physical increased after the activity (38.63 ± 38.86 to 75.00 ± 41.83; *p* = 0.03).
							Activity evaluation	Emotional words increased after the activity (1.40 ± 0.55 to 2.40 ± 0.55; *p* = 0.03). Amount of speech increased after the activity (1.20 ± 0.45 to 2.50 ± 0.55; *p* = 0.04). Satisfaction increased after the activity (1.60 ± 0.55 to 3.00 ± 0.00; *p* = 0.04).
							Salivary Chromogranin A	CgA decreased after the activity (1.14 ± 0.63 to 0.94 ± 0.74; *p* < 0.01).
	Paro	Before after study [32]	12	77.5 ± 7.3	11(91.6)	Elderly residents MMSE 15 to 29	Urinary test (17-KS-S values and the ratios of 17-KS-S/17-OHCS)	The hormone value of 17-KS-S and the 17-KS-S/17-OHCS ratio improved after the activity. 17-KS-S: 1.00 ± 0.51 to 1.41 ± 1.09 (*p* < 0.05) 17-KS-S/17-OHCS: 0.18 ± 0.08 to 0.26 ± 0.09 (*p* < 0.05)
		Before after study [27]	13	77.5 ± 7.4	11(84.6)	Elderly residents in a care house MMSE 15 to 29	Density of objective social network	The density was increased after the activity. 2nd floor: 0.26 to 0.29 3rd floor: 0.12 to 0.14
							Average time spent/day	The average of time spent by the participants was increased after the activity. 2nd floor: 5:27:02 to 8:52:00 3rd floor: 1:22:46 to 2:31:14
		Before after study [33]	29 - Elderly 23 - Nurses 6	Elderly 73 to 93 Nurses N/A	Elderly 23(100) Nurses 6(100)	Elderly people and nurses at a day service center	Facial expression (Face scale)	Scores increased through interaction with Paro, and scores were unchanged after the activity.
							Questionnaires concerning moods (POMS)	Scores of the question item “vigorous” after the activity increased compared with those before the activity (*p* < 0.05).
							Urinary tests (17-KS-S values and the ratios of 17-KS-S/17-OHCS)	The values and ratios were increased after the activity. Paro improved the ability of the elderly to recover from stress.
Humanoid agent	Relational agent	Non RCT [23]	21(8/8)(5 drop) - Relational 8 - Standard of care 8	I: 73.8 C: 74.2	I: 8(100) C: 5(63)	Elderly people No significant cognitive impairment English speaking ability	Loneliness (UCLA loneliness scale)	There were no significant differences (*p* > 0.05).
							Well-being (Satisfaction with life scale)	There were no significant differences (*p* > 0.05).
							Perceptions of relational agent	Reviews of the relational agent were mostly positive.
							Daily recorded steps	Increase per week in mean steps walked of the intervention group was more than the control group (I/C: 411.1/83.9; *p* = 0.004).
							Usage	Intervention group indicated that they would like to continue using the system.
							Usability	It was easy to use. The elderly felt that they and the agent understood each other.
	Conversational agent	Mixed methods [22]	14 (2 drop) - Proactive 7 - Passive 7	N/A	11(78.5)	Age ≥ 55 Living alone No significant depressive symptoms	Loneliness (UCLA loneliness scale)	Proactive group had a greater reduction in loneliness than the passive group (*p* = 0.13).
							Comport Satisfaction Happiness	Proactive group improved affective response toward the system than the passive group (*p* < 0.05). Comfort: 4.59 ± 0.80 to 4.33 ± 0.85 Satisfaction: 3.95 ± 1.08 to 3.14 ± 1.26 Happiness: 3.89 ± 0.90 to 3.26 ± 1.26
							Open-ended feedback	Participants appreciated when the agent induced positive affect through comforting statements. The agent acted as a friend.
Mobile robot	Assistive telepresence robot	Mixed methods [25]	35 - Elderly 30 - Caregivers 5	Elderly 64 Caregiver 40	Elderly 12(46.1) Caregiver N/A	Elderly people and caregivers in a private elderly care center	Semi-Structured Interviews	Most elderly people and caregivers were willing to use the robot in their everyday life.
		Mixed methods [28]	35 - Elderly 30 - Caregivers 5	Elderly 71 Caregiver N/A	Elderly 13(43) Caregiver N/A	Professional caregivers and elderly people in nursing home No severe disability problems	Perceived usefulness	Most elderly and caregivers perceived the robot as useful. Vital signs (Elderly/Caregiver: 3.2 ± 1.24/4.6 ± 0.55; *p* = 0.05) Reminder (Elderly/Caregiver: 3.1 ± 1.18/4.6 ± 0.55; *p* = 0.05) Video conference application (Elderly/Caregiver: 4.06 ± 0.98/3.0 ± 0.71; *p* = 0.05)
							Perceived ease of use	Navigation (3.0/3.2) was perceived as most difficult to use by both groups. Video conference application (4.4/4.8) was perceived as most easy to use by both groups.
Exercise game	Wii exergame developed for study	Non RCT [12]	30(10/10/10) - Exergame 10 - Traditional exercise 10 - Care as usual 10	I: 71 ± 6.58 C: 71.6 ± 5.15 C: 71.4 ± 8.46	I: 7(70) C: 8(80) C: 6(60)	Community-dwelling elderly people (Age ≥ 65) Ability to perform basic exercise No serious physical or cognitive disorders	Loneliness (Short form of ULS)	There were no significant differences (*p* = 0.129). I: 16.0 ± 3.27 to 12.9 ± 1.29 C: 14.8 ± 4.57 to 14.4 ± 1.29 C: 14.0 ± 6.60 to 15.2 ± 1.29
							Life satisfaction (SWLS)	There were no significant differences (*p* = 0.234). I: 27.0 ± 3.83 to 27.6 ± 1.64 C: 25.6 ± 6.62 to 29.2 ± 1.64 C: 28.6 ± 5.95 to 27.7 ± 1.64
							Exercise enjoyment (PACES)	Scores of exergame group had higher than those in other groups. I/C/C: 24.4 ± 0.65/22.3 ± 0.65/22.0 ± 0.65 (*p* > 0.05)
							Self-efficacy (GSE)	Scores of exergame group had lower than those in other groups (*p* = 0.283). I: 16.4 ± 2.84 to 14.5 ± 1.01 C: 17.0 ± 4.03 to 16.8 ± 1.01 C: 17.8 ± 4.34 to 18.4 ± 1.01
Interpersonal communication	iPad-based communication app	Before after study [36]	21 (9 drop)	82.5	8(66.6)	Residents at a retirement home No dementia	Social support (Duke social support scale) Loneliness (UCLA loneliness scale)	There were no significant changes (*p* > 0.05).
							Interview	The tool was portable, convenient, and simple. The app usage increased participants’ positive mood, self-efficacy, and comfort with technology.
Online social platform	LI-CBT	RCT [20]	32(13/16) (3 drop) - Intervention group 13 - Usual care 16	74.93 ± 6.41	I: 13(86.7) C:13(76.5)	Elderly people in residential care facilities (Age ≥ 60) Intact cognition on the SMCC Friendship scale ≤ 15 DJGLS 2 to 6 WHO-5 < 13	Loneliness (DJGLS)	There were significant decreases. I: 3.53 ± 1.3 to 1.38 ± 1.33 (*p* < 0.001) C: 3.59 ± 1.23 to 4.00 ± 1.32 (*p* = 0.064)
							Mental well-being (WHO-5)	There were no significant changes. I: 15.07 ± 6.87 to 16.54 ± 4.54 (*p* = 0.341) C: 17.24 ± 3.35 to 16.47 ± 4.00 (*p* = 0.591)
							Social cognition (YSQ-SF)	There were significant decreases. I: 83.53 ± 19.3 to 52.62 ± 15.99 (*p* = 0.008) C: 73.82 ± 29.05 to 78.00 ± 14.77 (*p* = 0.275)
							Usage	There were significant increases. 1 month later, there was a significant reduction.
	PRISM	RCT [19]	300(105/119) (56 drop) - PRISM 105 - Binder 119	I: 76.9 ± 7.3 C: 75.3 ± 7.4	I:119(79.3) C:115(76.7)	Age ≥65 Living alone in independent housing Speaking English At least 20/60 vision Having minimal computer/Internet use Not employed or volunteering more than 5 hr/week Spending more than 10 hr/week at a senior center Having permission on the basis of the results of the FULD MMSE ≥ 26	Social isolation (Friendship scale)	At 6 months, PRISM participants had a significant decline in social isolation (*p* < 0.01).
							Loneliness (UCLA loneliness scale)	At 6 months, PRISM participants had a significant decline and maintained at 12 months (*p* < 0.01).
							Social support (Interpersonal support evaluation list)	At 6 months, PRISM participants had a significant increase and maintained at 12 months (*p* < 0.01).
							Changes in health-related well-being (SF-36)	PRISM participants reported a greater increase (*p* < 0.05).
							Changes in attitudes toward technology	PRISM participants reported greater increases in computer comfort, interests, and efficacy (*p* < 0.01).
							Perceptions of the usefulness and usability (Technology acceptance questionnaire)	Participants found PRISM useful and easy to use. The most used features were E-mail, the Internet, and Games.
							Perceived vulnerability (Perceived vulnerability scale)	There were decreases in PRISM group (*p* < 0.001).
	LEAP	RCT [26]	75(48/22) (5 drop) - LEAP 48 - Usual care 22	I: 60.9 ± 3.4 C: 62 ± 3.9	I: 38(76) C: 19(76)	Retirement age Retiring in the last two years Planning to retire in the next two years Ability to access to the Internet Speaking English No severe mental health conditions Blood pressure < 180/110mmHg CESD < 20	Food consumption measures	There were small reductions in body weight (I/C: −0.6/−0.3kg) and waist circumference (I/C: −0.9/−0.4cm). Mediterranean diet score of Intervention group was higher (4.7 to 4.6) than the usual care group (3.8 to 3.8).
							Physical activity measures	For all physical activity measures, outcomes were similar for both groups.
							Interview	Participants regarded as addressing key concerns through retirement transitions. The contents were acceptable and reinforced the need for an intervention.

ICT = Information communication technology; AIBO = Artificial Intelligence Robot; RCT = Randomized controlled trial; MMSE = Mini-Mental State Examination; UCLA loneliness scale = University of California-Los Angeles Loneliness Scale; MLAPS = Modified the Lexington Attachment to Pets Scale; AOK loneliness scale = Ando, Osada, and Kodama Loneliness Scale; HR-QoL = Health-related quality of life; CgA = Salivary Chromogranin A; 17-KS-S = 17-ketosteroid sulfate; 17-KS-S/17-OHCS = 17-ketosteroid sulfate/17-hydroxycorticosteroids; POMS = The Profile of Mood States; Non RCT = Non-randomized controlled trial; Short form of ULS = Short Form of University of California-Los Angeles Loneliness Scale; SWLS = Satisfaction with Life Scale; PACES = Physical Activity Enjoyment Scale; GSE = General Self-Efficacy Scale; LI-CBT = Low-intensity Cognitive Behavior Therapy; SMCC = Subjective Memory Complaint Clinical; DJGLS = De Jong Gierveld Loneliness Scale; WHO-5 = World Health Organization-Five Well = Being Index; YSQ-SF = Young Schema Questionnaire; PRISM = The Personal Reminder Information and Social Management; FULD = Fuld Object Memory Evaluation; SF-36 = MOS 36-Item Short-form Health Survey; LEAP = Living, Eating, Activity, and Planning through retirement; CESD = Centre for Epidemiologic Studies Depression Scale.

## Appendix A. Search Term

**Population**	**Intervention**	**Outcome**
“The elderly” *OR* “elderly people” *OR* “older people” *OR* “elderly” *OR* “elderly person” *OR* “older frail adults” *OR* “seniors” *OR* “older adults” *OR* “aging population” *OR* “elderly population” *OR* “aging” *OR* “exp. aging/” *AND*	“information communication technology” *OR* “ICT” *OR* “social media” *OR* “exp. social media/” *OR* “telepresence robot” *OR* “robotic assistants” *OR* “assistive robotics” *OR* “rehabilitation robots” *OR* “exp. rehabilitation robot/” *OR* “mobile robotic systems” *OR* “robotic assistive technologies” *OR* “service robot” *OR* “virtual reality and social media *OR* virtual reality-based application” *OR* “rehabilitative applications” *OR* “VR-based social application” *OR* “virtual reality cognitive training programs” *OR* “video conference application” *OR* “social application” *OR* “technology applications” *OR* “mobile instant messaging” *OR* “online platforms” *OR* “social platform” *OR* “online service platform” *OR* “online social support tool” *OR* “social media platforms” *OR* “active video game” *OR* “persuasive video gaming” *OR* “video game role-play” *OR* “persuasive social impact game” *OR* “computer system” *OR* “exp. computer system/” *OR* “living in network connected communities” *OR* “exergames” *OR* “the Personal Reminder Information and Social Management system (PRISM)” *OR* “PRISM Buddy” *OR* “mHealth-supported intervention” *OR* “WhatsApp” *OR* “Social Bike application” *OR* “Will Sports game package” *AND*	“social isolation” *OR* “exp. social isolation/” *OR* “social support” *OR* “exp. social support/” *OR* “social connection” *OR* “social connectedness” *OR* “loneliness” *OR* “exp. loneliness/” *OR* “social interaction” *OR* “exp. social interaction/” *OR* “well-being” *OR* “exp. wellbeing/” *OR* “social ties” *OR* “social connectivity” *OR* “the quality of elderly life” *OR* “physical and cognitive training” *OR* “health care”
